# Supplementary material for: Identification of photoperiod-induced specific miRNAs in the adrenal glands of Sunite sheep (Ovis aries)
Source: Front Vet Sci. 2022 Jul 22;9:888207. doi: 10.3389/fvets.2022.888207 (PMC9354845; doi:10.3389/fvets.2022.888207)
Supplement: Supplementary file 1 [file Data_Sheet_1.ZIP › Supplementary file/miRDeep2 file.zip!/miRDeep2 file/Know_Structure/SP42A1/oar-mir-10a.pdf]

miRBase precursor : oar-mir-10a
Total read count : 96496
oar-miR-10a read count : 95940
remaining reads : 556

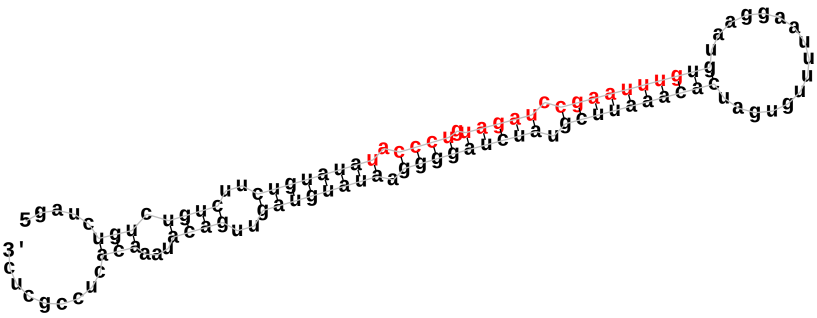

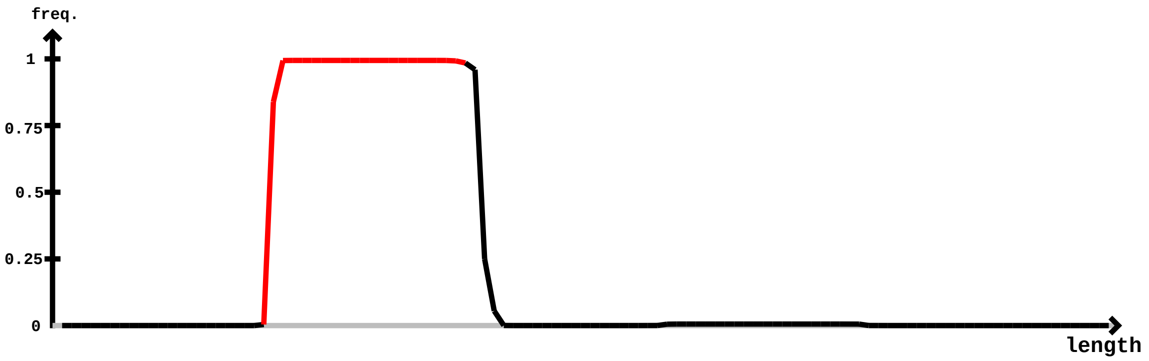


```
                        oar-miR-10a
5'- gaucugucugucuucuguauauacccuguagauccgaauuuguguaaggaauuuugugaucacaaauucguaucuaggggaauauguaguugacauaaacacuccgcuc   -3'    exp
    ....(((.((((..((((((((.((((.(((((.((((((((((...............)))))))))).))))))))).))))))))..))))...)))).........   reads   mm   sample
    .aucugucugucuucuAuaua........................................................................................   1       1    seq
    ....................auacccuguagauccgaa.......................................................................   1       0    seq
    ....................auacccuguagauccgaauu.....................................................................   1       0    seq
    ....................auacccuguagauccgaauuu....................................................................   17      0    seq
    ....................auacccuguagauccgaauuug...................................................................   54      0    seq
    ....................auacccuguagauccgaauuugu..................................................................   296     0    seq
    ....................auacccuguagauccgaauuugug.................................................................   17      0    seq
    ....................auacccuguagauccgaauuugugu................................................................   2       0    seq
    .....................uacccuguagauccgaau......................................................................   20      0    seq
    .....................uacccuguagauccgaauu.....................................................................   131     0    seq
    .....................uacccuguagauccgaauuu....................................................................   655     0    seq
    .....................uacccuguagauccgaauuug...................................................................   2117    0    seq
    .....................uacccuguagauccgaauuugu..................................................................   61280   0    seq
    .....................uacccuguagauccgaauuugug.................................................................   13272   0    seq
    .....................uacccuguagauccgaauuugugu................................................................   3035    0    seq
    .....................uacccuguagauccgaauuuguguа...............................................................   15      0    seq
    .....................uacccuguagauccgaauuuguguaa..............................................................   1       0    seq
    ......................acccuguagauccgaau......................................................................   2       0    seq
    ......................acccuguagauccgaauu.....................................................................   26      0    seq
    ......................acccuguagauccgaauuu....................................................................   79      0    seq
    ......................acccuguagauccgaauuug...................................................................   261     0    seq
    ......................acccuguagauccgaauuugu..................................................................   6879    0    seq
    ......................acccuguagauccgaauuugug.................................................................   5499    0    seq
    ......................acccuguagauccgaauuugugu................................................................   2228    0    seq
    ......................acccuguagauccgaauuugugua...............................................................   8       0    seq
    .......................cccuguagauccgaauuugu..................................................................   11      0    seq
    .......................cccuguagauccgaauuugug.................................................................   25      0    seq
    .......................cccuguagauccgaauuugugu................................................................   5       0    seq
    ........................ccuguagauccgaauuugu..................................................................   3       0    seq
    ............................................uaaggaauuuugugauca...............................................   1       0    seq
    ...........................................................ucacaaauucguaucuagggga............................   1       0    seq
    ...........................................................ucacaaauucguaucuaggggaa...........................   1       0    seq
    ............................................................cacaaauucguaucuaggggaau..........................   2       0    seq
    .............................................................acaaauucguaucuaggggaau..........................   17      0    seq
    ..............................................................caaauucguaucuagggga............................   9       0    seq
```

oar-miR-10a

```
gaucugucugucuucuguauauacccuguagauccgaauuuguguaaggaauuuugugaucacaaauucguaucuaggggaauauguaguugacauaaacacuccgcuc
..............................................................caaauucguaucuaggggaa...........................    9     0     seq
..............................................................caaauucguaucuaggggaau..........................    454   0     seq
..............................................................caaauucguaucuaggggaaua.........................    7     0     seq
..............................................................caaauucguaucuaggggaauaug.......................    1     0     seq
...............................................................aaauucguaucuaggggaa...........................    1     0     seq
...............................................................aaauucguaucuaggggaau..........................    45    0     seq
...............................................................aaauucguaucuaggggaauau........................    2     0     seq
................................................................aauucguaucuaggggaaua.........................    1     0     seq
................................................................aauucguaucuaggggaauau........................    4     0     seq
```
